# Induction Cisplatin, Docetaxel, and 5-Fluorouracil Versus Platinum Doublet in Human Papillomavirus-Associated Oropharyngeal Cancer

**DOI:** 10.3390/v18040446

**Published:** 2026-04-08

**Authors:** Ashwin Ganti, Patrick W. McGarrah, Harry Fuentes Bayne, Casey Fazer-Posorske, Binav Baral, Daniel J. Ma, Scott C. Lester, David M. Routman, Michelle A. Neben-Wittich, Jessica M. Wilson, Daniel L. Price, Eric J. Moore, Kendall K. Tasche, Katie M. Van Abel, Linda X. Yin, Nathan R. Foster, Katharine Price

**Affiliations:** 1 Division of Hematology-Oncology, University of California San Diego, San Diego, CA 92037, USA; ashwinganti7@gmail.com (A.G.); fuentes-bayne.harry@mayo.edu (H.F.B.); fazer.casey@mayo.edu (C.F.-P.); 2 Department of Oncology, Mayo Clinic, Rochester, MN 55905, USA; mcgarrah.patrick@mayo.edu (P.W.M.); baral.binav@mayo.edu (B.B.); 3 Department of Radiation Oncology, Mayo Clinic, Rochester, MN 55905, USAlester.scott@mayo.edu (S.C.L.); routman.david@mayo.edu (D.M.R.); neben.michelle@mayo.edu (M.A.N.-W.); wilson.jessica4@mayo.edu (J.M.W.); 4 Department of Otolaryngology-Head and Neck Surgery, Mayo Clinic, Rochester, MN 55905, USA; price.daniel@mayo.edu (D.L.P.); moore.eric@mayo.edu (E.J.M.); tasche.kendall@mayo.edu (K.K.T.); vanabel.kathryn@mayo.edu (K.M.V.A.); yin.linda@mayo.edu (L.X.Y.); 5 Division of Biomedical Statistics and Informatics, Mayo Clinic, Rochester, MN 55905, USA; foster.nathan@mayo.edu

**Keywords:** oropharyngeal cancer, human papillomavirus, HPV, de-escalation, oropharynx cancer, induction chemotherapy, platinum doublet

## Abstract

Management of locoregionally advanced human papillomavirus-positive oropharyngeal squamous cell carcinoma (HPV(+) OPSCC) can include induction chemotherapy followed by definitive chemoradiation. The standard induction regimen of cisplatin, docetaxel, and 5-fluorouracil (TPF) is associated with high toxicity. Given the chemosensitivity of HPV(+) OPSCC, platinum doublets are frequently used as induction therapy with potentially less toxicity. This retrospective study aimed to compare outcomes between treatment-naive HPV(+) OPSCC patients receiving induction TPF and those receiving an induction platinum doublet. Data collected included tumor characteristics, response after chemoradiation, hospitalization rates, and overall survival (OS). Fifty-five patients (18 TPF and 37 platinum doublet) were included. There was no significant difference in response after completion of definitive chemoradiation (TPF: CR 83.3%, PR 5.6%, progression or metastasis 11.1% vs. platinum doublet: CR 75.7%, PR 16.2%, progression or metastasis 8.1%; *p* = 0.5241). There were also no differences in hospitalizations for adverse events (38.9% in TPF vs. 40.5% in platinum doublet, *p* = 0.907) or recurrence (11.1% in TPF vs. 2.7% in platinum doublet, *p* = 0.198). The 5-year OS was 84.6% in the TPF group and 81.5% in the platinum doublet group (*p* = 0.581). Induction platinum doublet regimens offer comparable OS, response, and hospitalization rates to TPF in locally advanced HPV(+) OPSCC. Induction with a platinum doublet may be a viable de-escalation strategy for patients who are not candidates for TPF.

## 1. Introduction

Human papillomavirus (HPV)-positive oropharyngeal squamous cell carcinoma (HPV(+) OPSCC) is the most common type of head and neck cancer and is defined by the presence of high-risk types of HPV in tumor cells, predominantly HPV type 16, which is a strong and independent biomarker for prognosis [1]. HPV(+) OPSCC is very responsive to radiation and chemotherapy and has an improved prognosis compared with HPV-negative OPSCC [2]. Treatments of locally advanced HPV(+) OPSCC commonly include tumor resection followed by adjuvant radiation with or without cisplatin chemotherapy or definitive chemoradiation with cisplatin [3]. Despite the overall good prognosis for patients with HPV(+) OPSCC, approximately 20% of patients develop recurrence over time, with the majority presenting with distant metastatic disease, making escalation of systemic therapy appealing [2,4,5].

Induction chemotherapy followed by definitive radiation or chemoradiation is a known treatment strategy for locally advanced head and neck squamous cell carcinoma (HNSCC). However, the role of induction therapy in locally advanced HNSCC remains controversial, as three phase 3 randomized trials, although limited by accrual and power, did not demonstrate an overall survival benefit [6,7,8]. In contrast, the potential benefit of induction therapy was highlighted by a phase II-III study published in 2017 by the GSTTC Italian Study Group; this trial analyzed the role of cisplatin, docetaxel, and 5-fluorouracil (TPF) in patients with locally advanced head and neck cancer and identified benefits in overall survival and progression-free survival at 3 years [9]. A meta-analysis performed by Zhang et al. showed significant reduction in rates of distant metastases with induction chemotherapy compared to concurrent chemoradiation, albeit without an overall survival benefit [10]. As a result, induction chemotherapy remains a category 3 NCCN recommendation, considered in scenarios for patients at high risk of distant failure, such as those with large tumors, high nodal burden, rapidly progressive disease, or significant symptoms from disease [3,11].

Two landmark clinical trials established standard practices for safe administration of TPF as induction therapy for locally advanced HNSCC. The TAX 323 study, completed in 2007, utilized four cycles of docetaxel (75 mg/m^2^), cisplatin (75 mg/m^2^), and 5-fluorouracil (5-FU) (750 mg/m^2^) [12]. In contrast, the TAX 324 protocol utilized three cycles with higher doses of cisplatin (100 mg/m^2^) and 5-FU (1000 mg/m^2^), while maintaining the same dose of docetaxel (75 mg/m^2^) [13]. The TAX 323 and 324 trials, including long-term analyses, demonstrated that in patients with locally advanced HNSCC who are candidates for induction therapy, the addition of docetaxel to cisplatin and 5-fluorouracil improved overall survival, progression-free survival, locoregional control, and long-term survival benefit [12,13,14]. Following these studies, TPF became accepted as the standard choice of induction therapy for patients with locally advanced HNSCC. However, TPF is associated with significant toxicity, notably renal toxicity and cytopenia, leading to interest in induction chemotherapy regimens with less toxicity [15,16]. A 2014 investigation by Herman et al. compared induction with carboplatin and paclitaxel to induction with TPF in patients with locally advanced HNSCC without stratification by HPV status and demonstrated that the platinum doublet regimen yielded similar locoregional control and progression-free survival at 1 year while having less renal toxicity [17]. Due to the chemosensitivity of HPV(+) OPSCC, platinum doublets are often used as induction therapy in order to minimize toxicity in patients who are likely to have long-term survival after cancer treatment. Our preferred induction chemotherapy regimen for patients with HPV(+) OPSCC is carboplatin and paclitaxel due to ease of administration and overall tolerance; in select younger patients, cisplatin and docetaxel have been used successfully as well. Although prior TPF has been compared to platinum doublet in an unselected, HPV-agnostic population of patients with locally advanced HNSCC, there has been no investigation to date comparing these induction regimens specifically for locally advanced HPV(+) OPSCC [16]. In this retrospective study, we compare outcomes for patients with locally advanced HPV(+) OPSCC who received standard TPF induction chemotherapy followed by curative-intent radiation or chemoradiation with those who received a platinum doublet induction regimen.

## 2. Materials and Methods

Patients diagnosed with treatment-naïve HPV(+) OPSCC between 1 January 2010 and 31 December 2021 at any Mayo Clinic site (Rochester, MN, USA; Scottsdale, AZ, USA; Jacksonville, FL, USA) were identified through an institutional REDCap database (REDCap 15.5.38., Vanderbilt University, Nashville, TN, USA) of patients with oropharyngeal cancer. Patients with large tumors (T3 or T4), high nodal burden, rapidly progressive disease, or significant symptoms from disease necessitating rapid treatment response were considered candidates for induction therapy at the discretion of the treating provider. High nodal burden was defined as N2 or N3 disease per AJCC 8th edition staging, multiple ipsilateral or bilateral lymph nodes, bulky lymphadenopathy (>3 cm), or involvement of the lower neck (levels IV or V). Patients who received induction regimens of TPF or a platinum doublet regimen (cisplatin or carboplatin with paclitaxel or docetaxel) were included in the analysis. Selection of induction regimen was at the discretion of the treating physician and was not standardized. Factors affecting treatment choice were patient age, performance status, comorbidities, organ function and anticipated tolerance; these variables were not consistently available in this retrospective dataset. The dosage ranges administered are as follows: cisplatin 60–75 mg/m^2^, docetaxel 60–75 mg/m^2^, 5-FU 600–1000 mg/m^2^/day, carboplatin AUC 4–5, and paclitaxel 150–175 mg/m^2^. Patients scheduled to undergo operative intervention and patients who presented with recurrence of oropharyngeal malignancy were excluded from the analysis.

Variables collected included: age, sex, history of cigarette smoking, race, primary site of malignancy, TNM staging (AJCC 8th edition), induction regimen, number of induction cycles planned, number of induction cycles administered, need for growth factor administration (filgrastim or pegfilgrastim), radiographic response after completion of induction chemotherapy based on descriptive reports by subspeciality radiologists, chemotherapeutic regimen administered concurrent with radiation, number of concurrent chemotherapy cycles planned, number of concurrent chemotherapy cycles received, response 3 months after completion of chemoradiation, toxicities during induction therapy, and, if applicable, cause of death. Toxicities during the definitive chemoradiation portion of treatment were not collected, as the focus of this study was on induction therapy. Only clinically significant toxicities that altered medical management were recorded; examples of these include cytopenias that prevented administration of a scheduled course of chemotherapy, neutropenic fever, or electrolyte abnormalities that required hospitalization. Treatment responses after induction therapy were evaluated after completion of 2 to 3 cycles depending on the treating provider; radiographic response was assessed using CT and/or PET/CT imaging as a part of routine clinical practice and interpreted by subspecialty radiologists. Response assessment was not standardized, and no central radiology review was performed.

Statistical analysis was performed using BlueSky Statistics 10.3.1 (BlueSky Statistics LLC, Chicago, IL, USA). Overall survival and event-free survival (censored for progression, recurrence, or death), stratified by induction chemotherapy regimen, were computed with a Kaplan-Meier analysis. The median follow-up time was calculated using the reverse Kaplan–Meier method. Categorical variables between treatment groups were compared using χ^2^ or Fisher’s exact tests based on expected cell counts. Statistical significance was established as *p* < 0.05. The Institutional Review Board reviewed the study and deemed it exempt from requirement for formal approval.

## 3. Results

A total of 55 patients were included in the study cohort, of which 18 received TPF and 37 received a platinum doublet regimen; 29 patients received carboplatin with paclitaxel, and 8 patients received cisplatin with docetaxel. Patient demographics are depicted in Table 1. The average age of the cohort was 61 years; most were males (84%), and the cohort was predominantly White (98%). Nearly half (47%) of patients were former smokers. By the 8th edition AJCC staging criteria, patients most commonly had T3 or T4 disease (51%), N2 nodal disease (69%), and were without distant metastasis (100%). There were no statistically significant differences in patient demographics or tumor characteristics between the two treatment groups.

**Figure 1:**
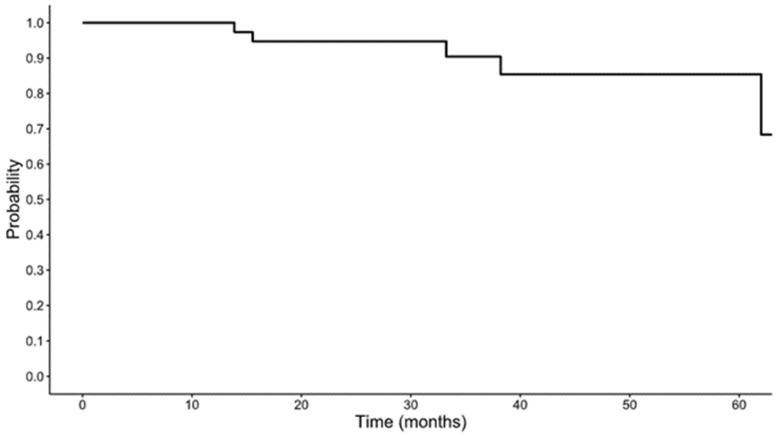
Overall survival for the whole cohort.

**Figure 2:**
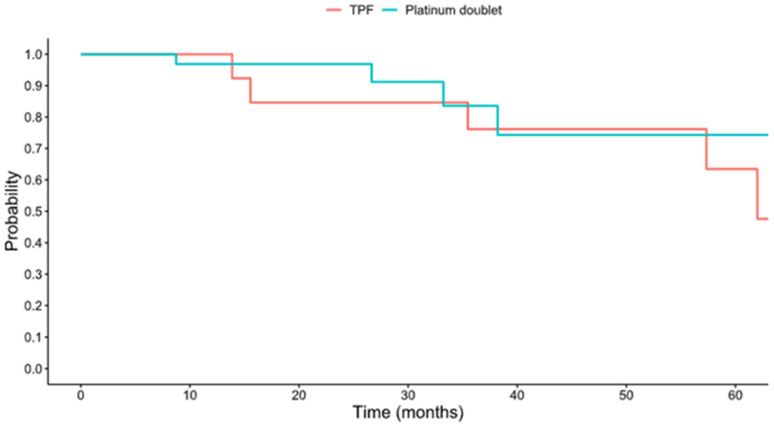
Overall survival by induction chemotherapy regimen.

**Figure 3:**
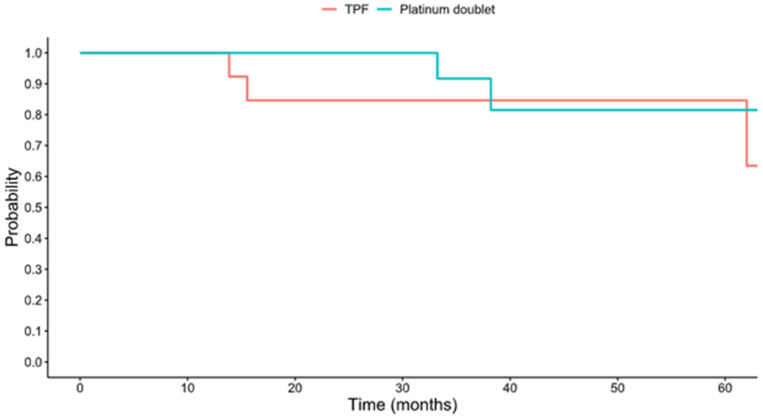
Event-free survival by treatment regimen.

The median follow-up time for the cohort was 26.6 months. The 5-year survival of the overall cohort was 85.4% (95% confidence interval [CI] 72.6–100%); this is depicted in Figure 1. The 5-year OS was 84.6% (95% CI 67.1–100%) in the TPF group and 81.5% (95% CI 61.1–100%) in the platinum doublet group (Figure 2), with no significant difference in OS between groups (*p* = 0.581). The hazard ratio of the platinum doublet group to TPF was 0.59 (95% CI 0.09–3.88). The 5-year event-free survival (EFS) was 63.5% (95% CI 39.5–100%) in the TPF group and 74.3% (54.1–100%) in the platinum doublet group (Figure 3); there was no significant difference in EFS (*p* = 0.665) between the groups.

All patients were planned for three cycles of induction therapy. There were no statistically significant differences in completion of induction therapy (77.7% for TPF vs. 91.2% for platinum doublet, *p* = 0.141), although there was a trend towards better completion in the platinum doublet group (Table 2). There was no statistically significant difference in the rate of complete response (CR) after completion of induction therapy (TPF CR 27.8% vs. platinum doublet CR 8.8%; TPF partial response [PR] 66.7% vs. platinum doublet PR 91.2%; *p* = 0.0567) or definitive chemoradiation (TPF: CR 83.3%, PR 5.6%, progression or metastasis 11.1% vs. platinum doublet: CR 75.7%, PR 16.2%, progression or metastasis 8.1%; *p* = 0.524). There were also no differences in hospitalizations for adverse events (38.8% in TPF vs. 40.5% in platinum doublet, *p* = 0.907). All patients went on to receive definitive chemoradiation with intensity-modulated radiation therapy (IMRT) to a total dose of 70 Gy in 35 fractions (2.0 Gy per fraction) over 7 weeks with concurrent weekly chemotherapy. Concurrent agents included weekly cisplatin (40 mg/m^2^) or weekly carboplatin (AUC 1.5–2), at the discretion of the treating physician. There were no differences in tumor recurrence (two recurrences with rate of 11.1% in the TPF group vs. one recurrence with a rate of 2.7% in the platinum doublet group, *p* = 0.198) between induction groups. The single recurrence in the platinum doublet group was a distant metastasis; the two recurrences in the TPF group were a local recurrence and regional recurrence. Toxicities of treatment are also depicted in Table 2; there was higher proportion of cytopenia in patients receiving platinum doublet (platinum doublet 45.9% vs. TPF 22.2%), but this was not statistically significant, nor were any other differences in recorded clinically significant toxicities. Significantly more patients who received TPF also received filgrastim or pegfilgrastim as growth factor support (TPF 83.3% vs. platinum doublet 24.3%, *p* < 0.001).

## 4. Discussion

This study is the first to compare induction chemotherapy with either a platinum doublet or TPF specifically in patients with newly diagnosed, locally advanced HPV(+) OPSCC receiving curative intent therapy. The first goal of this investigation was to determine rates of OS and EFS in the overall cohort and between treatment groups. We found that 5-year OS was 85.4% in the whole cohort, 84.6% in the TPF group, and 81.5% in the platinum doublet; there was no significant difference between these groups. Similarly, the 5-year EFS was 63.5% in the TPF group and 74.3% in the platinum doublet, which were also not statistically different.

The 5-year OS of 85.4% in our cohort is consistent with established benchmarks for locoregionally advanced HPV-positive OPSCC, including the 84.6% 5-year OS reported in the cisplatin arm of RTOG 1016 and the 81–88% 5-year OS across stages I–IVA in the ICON-S validation cohort [5,18]. These data confirm that outcomes with induction platinum doublet therapy are comparable to those expected for this favorable-prognosis population. These findings are also consistent with the retrospective analysis published in 2023 by Zhang et al. of 51 patients with HPV(+) OPSCC treated with induction chemotherapy, which demonstrated a 3-year OS of 92.4% [19]. Similarly, the low recurrence rate in our cohort aligns with published benchmarks for HPV(+) OPSCC, including 5-year locoregional and distant control rates of 92% and 89%, respectively [20]. Distant metastasis is the predominant failure mode in HPV-positive disease (pooled rate ~7%), with advanced T-stage conferring nearly 5-fold increased risk—supporting the rationale for induction chemotherapy in high-risk patients [21,22]. Our analysis suggests that utilizing a platinum doublet induction regimen results in similar 5-year OS and EFS as those of the standard of care TPF regimen.

The second goal was to explore rates of complete radiographic response between the two treatments groups. The TAX323 analysis, which compared induction with TPF to induction with cisplatin–fluorouracil noted significantly higher complete response rates in the TPF group (59% vs. 36% in the cisplatin–fluorouracil group), establishing TPF as the preferred standard over cisplatin–fluorouracil for induction therapy [6]. In our analysis, complete response rates after induction were lower (TPF 27.8%, platinum doublet 8.8%), although partial response rates were high (TPF 66.7%, platinum doublet 91.2%); these differences likely reflect our use of descriptive radiographic reports rather than standardized response criteria. After completion of induction and definitive chemoradiation, complete response rates exceeded 75% in both groups (TPF 83.3%, platinum doublet 75.7%; *p* = 0.524), reflecting the increased chemosensitivity of HPV-associated disease.

Our investigation also sought to explore rates of clinically significant toxicities and found no statistically significant difference in acute renal injury or cytopenia between the two groups; rates of hospitalization were also similar between the groups. Further, the higher proportion of cytopenia in the platinum doublet group could have been influenced by significantly greater use of growth factor support in TPF group (83.3% vs. 24.3%). There was a higher proportion of acute renal injury or electrolyte abnormality in patients receiving TPF (27.8% vs. 16.2% in the platinum doublet group), as well as a higher proportion of cytopenia in patients receiving platinum doublet (45.9% vs. 22.2% in the TPF group), but neither of these differences were statistically significant. These findings are consistent with prior published work reporting significantly increased renal toxicity in patients receiving TPF but could also reflect cisplatin exposure rather than treatment intensity alone, as carboplatin-based regimens have lower nephrotoxicity [17]. In their series, patients treated with TPF had significantly higher rises in creatinine during the course of induction chemotherapy, which altered the subsequent doses of therapy during concurrent chemoradiation. In addition, their analysis also did note significantly higher neutropenia in the group receiving a platinum doublet regimen. In our cohort, the lack of difference in clinically significant cytopenias is due to more patients in the TPF group receiving growth factor support that would have mitigated the risk of myelosuppression, which is a known risk of the triple cytotoxic regimen. Overall, our analysis does suggest that clinically significant toxicities between the two regimens are comparable, although this should be interpreted with caution. The overall burden of adverse events is likely underreported, and toxicity grading was not standardized. In clinical practice, TPF is viewed as a harder and more toxic therapy regimen and is often reserved for patients who are young and fit, whereas carboplatin and paclitaxel are often given to elderly patients and those with medical comorbidities. The possibility of selection bias remains a limitation, as treatment assignment was not randomized and likely reflected clinicians’ assessment of performance status, comorbidities and anticipated tolerance of patients. Further, clinical variables such as performance status and medical comorbidities could not be incorporated into the analysis due to variability in documentation. As such, firm conclusions about comparative toxicity from this retrospective study are not possible.

Notably, our study provides additional evidence for the use of a non-cisplatin induction regimen in patients with locally advanced HPV(+) OPSCC. The toxicities of cisplatin are well documented and include nausea, nephrotoxicity, neurotoxicity, and ototoxicity. Patients with pre-existing hearing loss, neuropathy, or chronic kidney disease are often not candidates for cisplatin therapy. Prior analyses have suggested that approximately one-third of patients who receive cisplatin developed nephrotoxicity; in addition, 30 to 50% of patients develop peripheral sensory neuropathy, with 10% developing disability secondary to this, and 40 to 80% of patients develop some degree of permanent sensorineural hearing loss from cisplatin [15,16]. Although further prospective analyses would be necessary to directly compare the two platinum doublets, our data offer a potential alternative to cisplatin for patients who require induction but are not good candidates for cisplatin.

In a broader context, these data align with the findings of several trials that have investigated changes in systemic therapy as de-escalation strategies for HPV(+) OPSCC. HPV(+) OPSCC is distinctly different from HPV-negative OPSCC in its molecular characteristics and prognosis, and in that it affects younger patients [1]. As a result, these patients often live longer with toxicity from any treatment, underscoring the importance of minimizing toxicity and understanding the role of de-escalation of therapy [23,24]. ECOG 1308 used induction chemotherapy and cetuximab to select patients for a decreased dose of radiotherapy. In this phase II trial, 77 patients received induction therapy with three cycles of weekly cisplatin, paclitaxel, and cetuximab, followed by radiation therapy, with reduced dosage of radiation being administered to those who demonstrated complete response after induction [25]. Patients who received a reduced dose of radiation demonstrated satisfactory clinical outcomes but improved swallowing and nutritional status. One salient aspect of the study is the utilization of response to induction chemotherapy as a guide to further treatment and potential dose-reduction, known as response-adapted de-escalation, which may allow for identification of favorable-risk populations and treatment de-escalation [26]. RTOG 1016 and the European DeEscalate trial both investigated the potential use of cetuximab instead of cisplatin along with definitive radiotherapy to spare patients toxicities of cisplatin, but the cetuximab arm was found to be inferior in both studies [18,27]. Our study supports the use of non-cisplatin induction regimens prior to radiotherapy as a potential de-escalation strategy, but prospective validation is needed.

There are several limitations of this analysis; this study was conducted as a retrospective review, and further prospective analysis in a clinical trial is warranted. Response assessment should be interpreted with caution, as measurement variability in response assessment in the absence of standardization and central radiology review may have occurred. In addition, the study was not powered to determine rates of toxicity, and this may have increased the probability of a type II error in the comparison of toxicities. Finally, the demographics of our patient cohort are relatively homogenous and may limit extrapolation of these results to patients of other races or age groups.

## 5. Conclusions

Patients with locally advanced HPV(+) OPSCC who received a platinum doublet regimen as induction therapy prior to definitive radiotherapy or chemoradiotherapy had similar OS, response after definitive chemoradiation, rate of hospitalizations, and rate of recurrence as patients who received induction TPF. Induction with a platinum doublet for locally advanced HPV(+) OPSCC appears to demonstrate similar observed outcomes to TPF and could be a de-escalation strategy for patients who are not candidates for TPF or to limit long-term cisplatin toxicities. These findings warrant further prospective validation given the limitations of our study design.

## Figures and Tables

**Table 1:** Patient demographics and characteristics.

	TPF (*n* = 18)	Platinum Doublet (*n* = 37)	*p*-Value
**Average age (range) in years**	60.67 (46–73)	61.8 (49–83)	0.140
**Male**	14 (77.7%)	32 (86.5%)	0.413
**Race**			0.482
White	18 (100%)	36 (86.5%)	
Native American	0 (0%)	1 (2.7%)	
**Smoking status**			0.936
Current smoker	3 (16.67%)	5 (13.5%)	
Former smoker	8 (44.4%)	18 (48.6%)	
Never smoker	7 (38.9%)	14 (37.8%)	
**Primary Site**			0.555
Tonsil	9 (50%)	14 (37.8%)	
Base of tongue	8 (44.4%)	18 (48.6%)	
Oropharynx, NOS	1 (5.55%)	5 (13.5%)	
**T**			0.989
1	3 (16.66%)	6 (16.2%)	
2	3 (16.66%)	7 (18.9%)	
3	3 (16.66%)	5 (13.5%)	
4	9 (50%)	19 (51.4%)	
**N**			0.122
0	0	1 (2.7%)	
1	1 (5.56%)	9 (24.3%)	
2	15 (83.3%)	23 (62.2%)	
3	2 (11.1%)	4 (10.8%)	
**M**			
0	18 (100%)	37 (100%)	
**Platinum doublet regimen**			
Carboplatin/paclitaxel		29	
Cisplatin/docetaxel		8	
**Growth factor support**			<0.001
Yes	15 (83.3%)	9 (24.3%)	
No	3 (16.7%)	28 (75.7%)	

**Table 2:** Treatment delivery and toxicities.

Outcome	TPF (*n* = 18)	Platinum Doublet (*n* = 37)	*p*-Value
**Completion of induction therapy**			0.141
Did not complete induction therapy	4 (22.2%)	3 (8.1%)	
Completed induction therapy	14 (77.8%)	34 (91.9%)	
**Completion of chemoradiation**			0.090
<5 cycles of chemotherapy	1 (5.6%)	9 (24.3%)	
≥5 cycles of chemotherapy	17 (94.4%)	28 (75.7%)	
**Post-induction response**			0.057
Partial response	12 (66.7%)	31 (83.8%)	
Near complete or complete response	5 (27.8%)	3 (8.1%)	
**Post-chemoradiation response**			0.524
Partial response	1 (5.6%)	6 (16.2%)	
Near complete or complete response	15 (83.3%)	28 (75.7%)	
Metastasis/progression	2 (11.1%)	3 (8.1%)	
**Hospitalization**			0.907
No hospitalization	11 (61.1%)	22 (59.5%)	
Hospitalization	7 (38.9%)	15 (40.5%)	
**Recurrence**			0.198
No recurrence	16 (88.9%)	36 (97.3%)	
Recurrence	2 (11.1%)	1 (2.7%)	
**Toxicity**			0.239
Acute kidney injury	5 (27.8%)	6 (16.2%)	
Cytopenia	4 (22.2%)	17 (45.9%)	
Ototoxicity	0 (0%)	4 (10.8%)	
Other toxicity	1 (5.6%)	2 (5.4%)	

## Data Availability

The data presented in this study are available on request from the corresponding author due to the retrospective nature of the study.

## Abbreviations

The following abbreviations are used in this manuscript:

HPV	Human papillomavirus
OPSCC	Oropharyngeal squamous cell carcinoma
OS	Overall survival
CR	Complete response
PR	Partial response
TPF	Cisplatin, docetaxel, infusional 5-fluorouracil
AJCC	American Joint Commission on Cancer
EFS	Event-free survival
